# *Toxoplasma gondii* Histone 4 Affects Some Functions of Murine Ana‐1 Macrophages In Vitro

**DOI:** 10.1111/jeu.12630

**Published:** 2018-05-17

**Authors:** Xinchao Liu, Xiaoyu Li, Qiangqiang Wang, Xiaoni Sun, Mingmin Lu, Muhammad Ehsan, Lixin Xu, RuoFeng Yan, XiaoKai Song, XiangRui Li

**Affiliations:** ^1^ College of Veterinary Medicine Nanjing Agricultural University Nanjing 210095 China

## Abstract

*Toxoplasma gondii* (*T. gondii*) is an obligate intracellular protozoan that can infect almost all nucleated cells. Histone proteins and DNA form the nucleosomes, which are the fundamental building blocks of eukaryotic chromatin. Histone 4 is an essential component of a histone octamer. In the present study, *T. gondii* histone 4 (TgH4) was cloned and the regulatory effect of TgH4 on murine macrophages was characterized. Bioinformatics analysis revealed that TgH4 was highly conserved in structure. Recombinant TgH4 (rTgH4) protein was identified by sera from rats experimentally infected with *T. gondii* and native TgH4 in the total soluble protein of *T. gondii* tachyzoites was recognized by polyclonal antibodies against rTgH4, as indicated by immunoblotting analysis. Immunofluorescence assay showed that TgH4 binds to macrophages. Following incubation with rTgH4, the toll‐like receptor 4 (TLR4) level of the macrophages was downregulated. Meanwhile, chemotaxis and the proliferation of macrophages were inhibited. However, rTgH4 can promote phagocytosis, apoptosis, and the secretion of nitric oxide, interleukin‐6, and tumor necrosis factor‐α from macrophages. Just 80 μg/ml rTgH4 can significantly elevate the secretion of interleukin‐10 and interleukin‐1β (*p *<* *0.05 and *p *<* *0.01). Viewed together, these outcomes indicated that rTgH4 can affect the functions of murine macrophages in vitro.

*TOXOPLASMA gondii* is a facultative and heteroxenous protozoan that can infect almost all warm‐blooded animals, including wild carnivores and marine mammals (Mazzariol et al. 2012; Sobrino et al. 2007). Overall, the infections of *T. gondii* in immunocompetent humans are asymptomatic; however, *T. gondii* infection during gestation will lead to abortion or congenital disease in its direct hosts (Duncanson et al. 2001; Moreno et al. 2012). Immunocompromised patients, especially those with deficient cellular immunity, may suffer from the reactivation of preexisting latent *T. gondii* infection (Ferreira and Borges 2002).

The immune response caused by *T. gondii* is sophisticated, complex, and individual. The predominant reaction of *T. gondii* infection in an immunocompetent host is considered to be cell‐mediated immunity (Denkers and Gazzinelli 1998). The macrophages and natural killer (NK) cells are the largest components in an immune response (Filisetti and Candolfi 2004), and macrophages have a crucial part in the removal of pathogens. The functions of macrophages in their defense against pathogens include the production of cytokines, such as interleukin‐12 (IL‐12), exerting microbicidal effector mechanisms, such as phagocytosis, and the production of NO (Aderem and Underhill 1999; Hunter et al. 1995; Wandurska‐Nowak 2004). In addition, apoptosis, proliferation, chemotaxis, and toll‐like receptors (TLRs), the major receptors for pathogen binding on the cell membrane of macrophages, also play a vital role in the defense against pathogens (Franken et al. 2016; Lopes et al. 2000; Toure‐Balde et al. 1996; Wujcicka et al. 2015).

A histone octamer wrapped in DNA forms nucleosomes, the fundamental unit of eukaryotic chromatin. Histone 4, one of the core histones, together with H2A, H2B, and H3, forms a histone octamer (Angelov et al. 2004). Consequently, histones play key roles in the support of the chromatin structure. Additionally, histone modifications in the chromatin‐mediated regulation of gene expression are a hot research area of epigenetic mechanisms (Rintisch et al. 2014). The well‐defined histone methylation marks include H3K4me3 and H3K27me3, connected with gene activation and repression, respectively (Barski et al. 2007).

The genome of *T. gondii* is predicted to encode the core histones. Posttranslational modifications (PTM) of histones play essential roles in epigenetic gene regulation. Proteomic studies revealed that many peptides of *T. gondii* histones undergo PTM which was associated with the activation or repression of genes (Nardelli et al. 2013). *T. gondii* histones were also engaged in epigenetic gene regulation. The expression of H2AX, a variant of H2A, can increase the generation of bradyzoites in vitro (Dalmasso et al. 2009). However, whether histones account for the regulation of host response remains to be elucidated. In this paper, potential effects of *T. gondii* histone 4 were investigated on murine macrophage phagocytosis, apoptosis, chemotaxis, and cytokines secretion. The results presented herein will provide significant insight into the effects of histones and further illuminate the biological roles of TgH4.

## Materials and Methods

### Animals

Eight‐week‐old female Sprague‐Dawley (SD) rats were bought from the Centre of Comparative Medicine, Yangzhou University (Yangzhou, China) and kept in a specific pathogen‐free environment. The evaluation was performed following the guidelines of the Animal Ethics Committee, Nanjing Agricultural University, China. All of the experimental protocols were authorized by the Science and Technology Agency of Jiangsu Province. The approval ID is SYXK (SU) 2010‐0005.

### Parasites and cell culture

The cell lines (Ana‐1 and Vero) and *T. gondii* RH strain were kept in the Laboratory of Veterinary Molecular and Immunological Parasitology, Nanjing Agricultural University, China.

Murine macrophages (Ana‐1) utilized in cellular function evaluations and Vero cells utilized to sustain *T. gondii* were cultured in Dulbecco's modified Eagle's medium (Gibco, New York City, NY) augmented with 10% dialyzed fetal bovine serum (Gibco) and 1% penicillin–streptomycin (Gibco) in a CO_2_ incubator (Thermo, Waltham, MA) at 37 °C.

### Bioinformatics analysis

Genedoc software (PSC, Pittsburgh, PA) was used to analyze sequence conservation, and the tertiary structure of TgH4 was predicted using SWISS‐MODEL approaches, which are accessible on the Internet (https://swissmodel.expasy.org/interactive).

### Molecular cloning of TgH4 and expression of recombinant TgH4 protein (rTgH4)

The open‐reading frame (ORF) of the *T. gondii* H4 gene was amplified from tachyzoites cDNA by PCR with the following primers, forward primer, 5′‐CCGGAATTCGAGTTACGCACATCCTGTCTTTTC‐3′, and reverse primer, 5′‐CCCAAGCTTCCTGTTTAGCTTCGCTTGTTCATT‐3′. Finally, the purified gene segments were cloned into the prokaryotic expression vector pET‐32a (+). After induction with IPTG (Sigma‐Aldrich, St Louis, MO), the rTgH4 protein fused to the 109 aa Trx. Tag thioredoxin with a C‐terminal His‐tag (fusion protein called further in the paper rTgH4) was purified using a Ni^2+^‐nitrilotriacetic acid (Ni‐NTA) column (GE Healthcare, Madison, WI) based on the company's directions (Zhang et al. 2014, 2016). Then, the pET‐32a vector Trx. Tag thioredoxin‐encoded protein without TgH4 fused to it (called further in the paper pET‐32a vector protein) was expressed to establish a control group for this evaluation.

### Polyclonal antibodies against rTgH4

To acquire the polyclonal antibodies against rTgH4, two rats were immunized with 250 μg rTgH4 protein emulsified with complete Freund's adjuvant (Sigma‐Aldrich). After 2 wk, a booster dose with 250 μg rTgH4 protein combined with Freund's incomplete adjuvant was given to the rats. Three more boosters with the same dose of rTgH4 were given to the rats in 1‐wk intervals. At 1 week following the last injection, sera were acquired. Two other rats were experimentally infected with *T. gondii* to acquire sera against *T. gondii*.

### Western blot

The polyclonal antibodies against rTgH4 and the sera from experimentally infected rats were used for immunoblotting analysis. *T. gondii* tachyzoites were sonicated on ice utilizing the sonication system pulse for 5 s on and 10 s off for 50 cycles. After that, the tubes were spun at 4 °C in a microcentrifuge at 10,000 *g* for 10 min. An ultrafiltration tube with 3 kDa molecular weight cutoff (Millipore, Bedford, MA) was used to condense the supernatant, and the condensed solution was utilized as the total soluble protein of *T. gondii* tachyzoites.

The total soluble protein of *T. gondii* tachyzoites and rTgH4 protein was separated by 10% SDS‐PAGE and the proteins were transferred to polyvinylidene fluoride (Millipore). Following blocking with 5% (w/v) skimmed milk powder in TBS (Tris‐buffer saline)‐Tween20 (TBST), the membranes were incubated with primary antibodies for 2 h at 37 °C (1:100 dilutions). The membranes were then rinsed thrice and incubated with horseradish peroxidase (HRP)‐conjugated goat anti‐rat IgG (Sigma‐Aldrich) at 37 °C for 1 h. Finally, a DAB Horseradish Peroxidase Color Development Kit (Beyotime, Shanghai, China) was utilized to identify the bound antibodies.

### Laser scanning confocal microscope

A laser scanning confocal microscope (PerkinElmer, Waltham, MA) was used to confirm the combination of TgH4 according to the previous study (Liu et al. 2017). Briefly, the murine macrophages were incubated with rTgH4 protein, pET‐32a vector protein, and phosphate‐buffered saline (PBS) in a 12‐well plate (Costar, Cambridge, MA). Then, macrophages were fixed with 4% paraformaldehyde in PBS, rinsed thrice in PBS containing 0.05% Tween‐20 (PBST) and finally incubated with BSA for 2 h at 37 °C. After that, rat anti‐rTgH4 sera (1:100 dilutions) were added and incubated at 4 °C overnight. Then, goat anti‐rat IgG antibody labeled with Cy3 (Beyotime) and DAPI was utilized. Finally, fluorescent mounting medium (Beyotime) was added and the cells were viewed via laser scanning confocal microscope (630× magnification).

### Proliferation and chemotaxis

All of the macrophages used for experiments were preincubated with rTgH4 protein at various concentrations (0, 5, 10, 20, 40, and 80 μg/ml) and the pET‐32a vector protein for 24 h, respectively. The proliferation of Ana‐1 cells was determined with a Cell Counting Kit‐8 (CCK‐8, Beyotime). After 24 h, macrophages were incubated with 10 μl CCK‐8 solution for another 2 h. Cell proliferation was then quantified according to the OD450 values established with a microplate spectrophotometer (BioRad, Hercules, CA).

Chemotaxis experiments were carried out with 24‐well plates (Costar, Cambridge, MA) and Millicell Hanging Cell Culture Inserts (Millipore). Chemoattractants were made to the desired concentrations and loaded into the lower wells of the 24‐well plates. Upper wells were filled with macrophages treated with proteins. The wells with macrophages and no rTgH4 protein loaded above the chemoattractants were set as the control groups and the wells with macrophages and no rTgH4 without the chemoattractants were set as the blank groups. The plates were incubated in a CO_2_ incubator (Thermo) at 37 °C for 12 h, and the migration rate of the macrophages was established.

### Flow cytometry

Macrophages diluted to 1 × 10^7^ cells/ml in PBS were preincubated with rTgH4 for 24 h at 37 °C, and then subsequently stained with an Annexin V‐FITC Kit (Miltenyi Biotec, Bergisch Gladbach, Germany), FITC‐dextran (Sigma‐Aldrich), and active CD284 (Biolegend, San Diego, CA), and analyzed by flow cytometry (BD Biosciences, San Jose, CA).

### Cytokines and NO secretion analysis

The cell supernatants were collected following 24 h of treatment with various concentrations (0, 5, 10, 20, 40, and 80 μg/ml) of rTgH4 protein and pET‐32a vector protein. The productions of tumor necrosis factor‐α (TNF‐α), interleukin‐1β (IL‐1β), interleukin‐6 (IL‐6), interleukin‐10 (IL‐10), and IL‐12 were quantified with Cytometric Bead Array (CBA) cell signaling flex sets (BD Biosciences). A Total Nitric Oxide Assay Kit (Beyotime) was utilized to investigate NO production.

### Statistical analysis

Data are shown as the means ± the standard deviation (SD). All of the data acquired from the above experiments were examined with Graphpad Prism 5.0 software (GraphPad Software, La Jolla, CA). Variations among groups were established as being significant at *p *<* *0.05.

## Results

### *Toxoplasma gondii* Histone 4 is highly conserved

**Figure 1:**
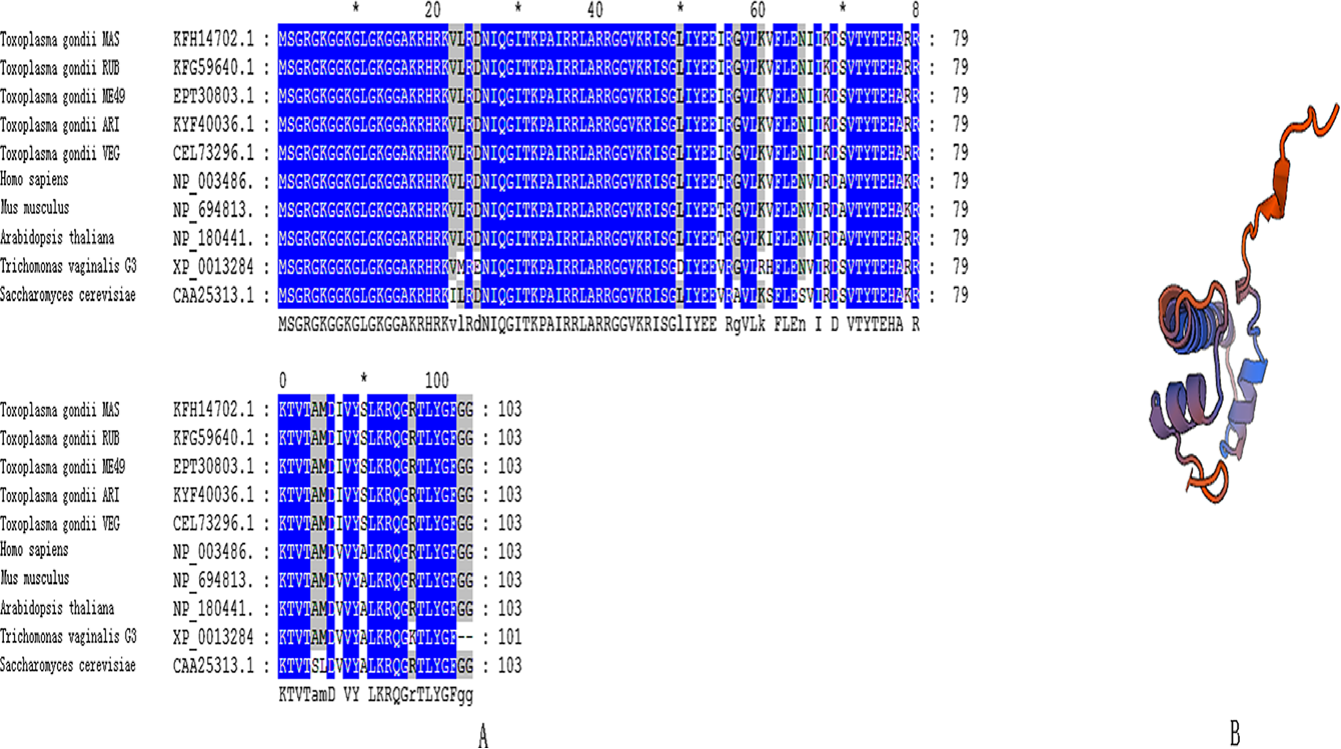
Multiple amino acid sequence alignment and a model of the tertiary structure of TgH4. (**A**) H4 amino acid sequence were aligned by Genedoc software. The amino acid sequences of histone 4 in different strains of *Toxoplasma gondii* were identical, and there was only a five amino acid difference between *T. gondii* and *Homo sapiens*. (**B**) A model of the tertiary structure of TgH4 was built using SWISS‐MODEL approaches and it matches the known H4 templates built before.

The amino acids of TgH4 were analyzed by Genedoc software (PSC, Pittsburgh, PA) and SWISS‐MODEL approaches. The results showed that the sequence of *T. gondii* H4 was highly conserved. The amino acid sequences of histone 4 in different strains of *T. gondii* were identical and there was only a difference of five amino acids between *T. gondii* and *Homo sapiens* (Fig. 1A). A model of the three‐dimensional structure was prepared and it matches the known H4 templates built before (Fig. 1B).

### TgH4 is an antigen protein with immunogenicity

**Figure 2:**
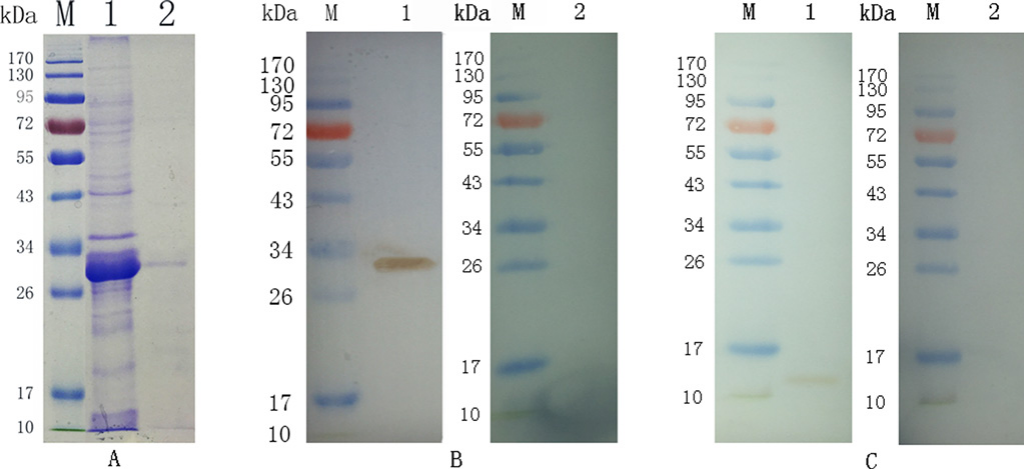
(**A**) Purification of rTgH4 shown on SDS‐PAGE. (Lane M): protein marker, (Lane 1): induction results of rTgH4 protein, (Lane 2): purified rTgH4 protein fused with pET‐32a vector protein. (**B**) Western blotting of rTgH4. (Lane M) protein marker; (Lane 1) recombinant protein TgH4 probed by serum from rats experimentally infected with *Toxoplasma gondii* as the primary antibody; (Lane 2) recombinant protein TgH4 probed by sera of normal rats as the primary antibody. (**C**) Western blotting of the total soluble protein of *T. gondii* tachyzoites. (Lane M) protein marker; (Lane 1) the total soluble protein of *T. gondii* tachyzoites probed by sera from rats immunized by rTgH4, and (Lane 2) the total soluble protein of *T. gondii* tachyzoites probed by sera of normal rats.

The molecular weight of TgH4 was 11.4 kDa, as estimated by DNAstar software (DNAstar, Madison, WI), and the rTgH4 (with its His‐Tag and fused pET‐32a vector protein) was about 32 kDa (Fig. 2A). The sera from rat experimentally infected *T. gondii* were utilized to identify the recombinant protein. The result showed a band of about 32 kDa, which indicated that TgH4 can induce the production of a specific antibody in the host. In addition, the sera against the recombinant protein were utilized to identify the protein in a total soluble extract of *T. gondii* tachyzoites. On the blot shown in Fig. 2C, it was identified as a band of about 12 kDa, in agreement with the molecular weight predicted from the sequence.

### rTgH4 binds to the murine macrophage surface

**Figure 3:**
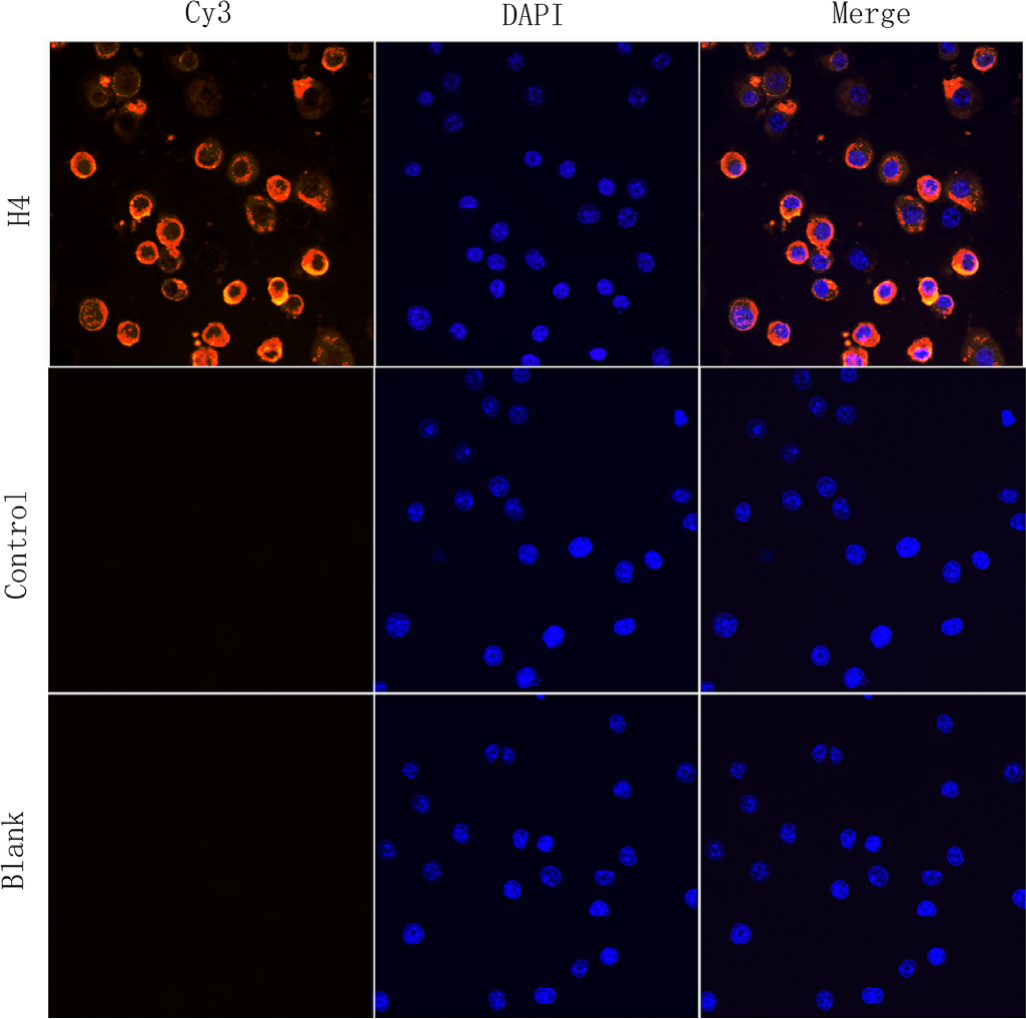
rTgH4 binds to murine macrophages. The nuclei of the macrophages were stained by DAPI (blue). Cy3‐conjugated secondary antibody was used to visualize the rTgH4 protein (red). Merged, overlapping the blue channels with red channels. No red fluorescence was noted in the PBS blank group or the pET‐32a vector protein control group.

The cells were incubated with rTgH4 to confirm if rTgH4 was a macrophage‐binding protein. The results of laser‐scanning confocal microscopy indicated that rTgH4 was binding to macrophages. The nuclei were revealed by blue fluorescence staining (DAPI), and the surface of the cells incubated with rTgH4 demonstrated red fluorescence staining (Cy3). Meanwhile, the control cells without rTgH4 showed no red fluorescence staining (Fig. 3).

### rTgH4 affects the TLR4 level of murine macrophages

**Figure 4:**
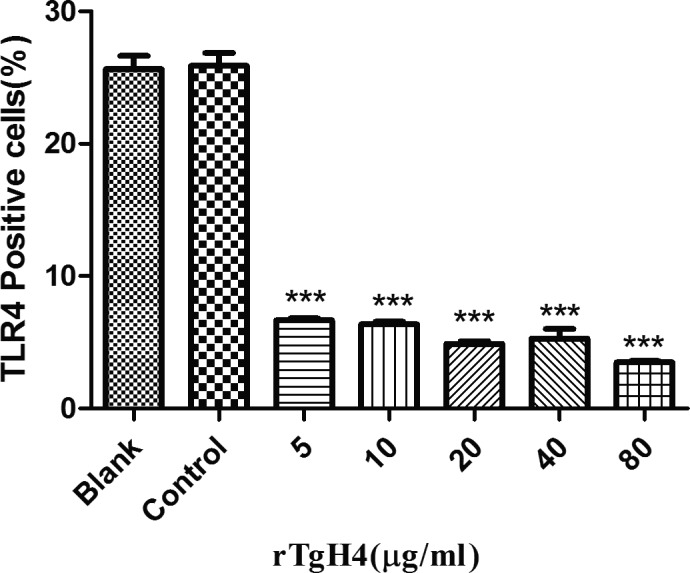
rTgH4 regulated the TLR4 level of the murine macrophages. The TLR4 level of the macrophages was detected by flow cytometry. In the blank group, the cells were treated with PBS, and in the control group, the cells were treated with pET‐32a vector protein. The data were indicative of three individual experiments (***p < 0.001).

The expression level of TLR4 on macrophages was detected by flow cytometry. No regulation of TLR4 level was observed in the control group, to which no rTgH4 was added, whereas TLR4 was shown to be downregulated in macrophages treated with rTgH4 (Fig. 4).

### rTgH4 inhibited the proliferation of murine macrophages

**Figure 5:**
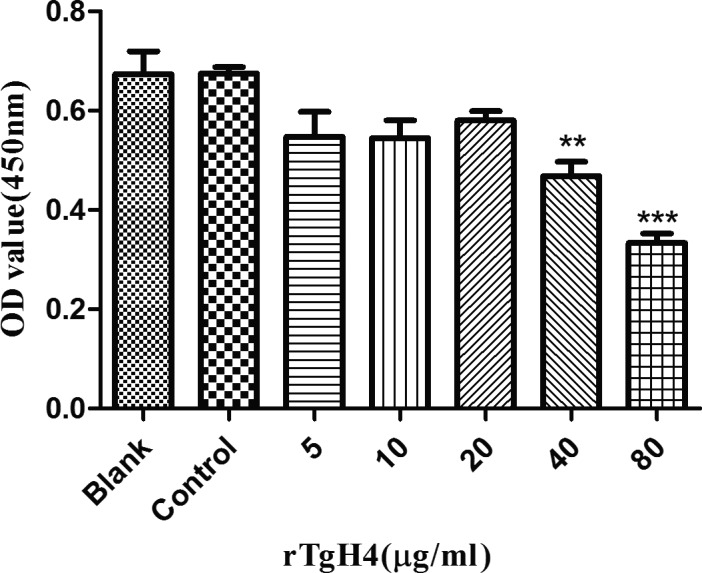
rTgH4 inhibited the proliferation of the murine macrophages. The proliferation assay was performed by CCK‐8. The OD450 value was measured to calculate the cell proliferation index. In the blank group, the cells were treated with PBS, and in the control group, the cells were treated with pET‐32a vector protein. The data were indicative of three individual experiments (**p < 0.01 and ***p < 0.001).

After treatment with CCK‐8, the OD450 values indicated that no significant difference was observed between the blank group treated with PBS and the control group treated with pET‐32a vector protein, while the proliferation of murine macrophages was inhibited in the groups incubated with 40 and 80 μg/ml rTgH4 (Fig. 5).

### rTgH4 promoted the phagocytosis of murine macrophages

**Figure 6:**
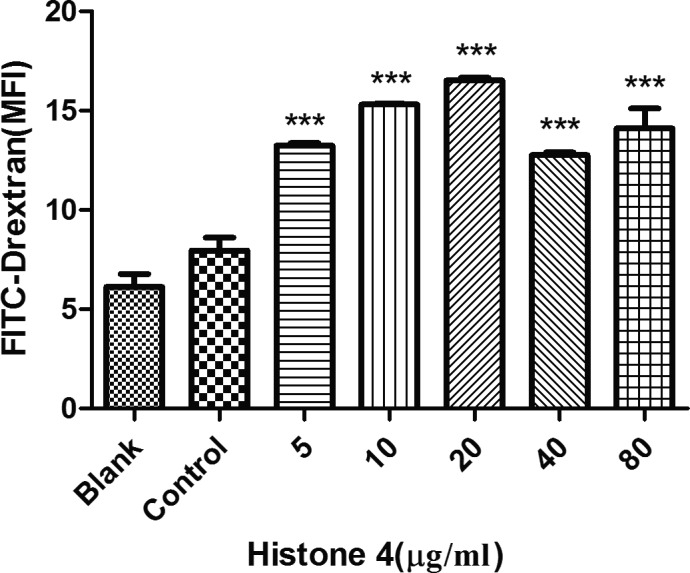
rTgH4‐induced phagocytosis of the murine macrophages. Phagocytosis was quantified by flow cytometry following incubation with 1 mg/ml FITC‐dextranin PBS at 37 °C for 1 h. The cell phagocytosis index was established based on the statistical data detailing the MFI (median fluorescence intensity) value. In the blank group, the cells were treated with PBS, and in the control group, the cells were treated with pET‐32a vector protein. The data were indicative of three individual experiments (***p < 0.001).

Flow cytometry assays were performed to study the effect of rTgH4 on the macrophage function of FITC‐dextran internalization. The results indicated that no difference was observed between the blank group treated with PBS and the control group treated with pET‐32a vector protein. The phagocytosis ability of the macrophages was significantly increased after the treatment with rTgH4 (Fig. 6).

### rTgH4 protein promoted the apoptosis of murine macrophages

**Figure 7:**
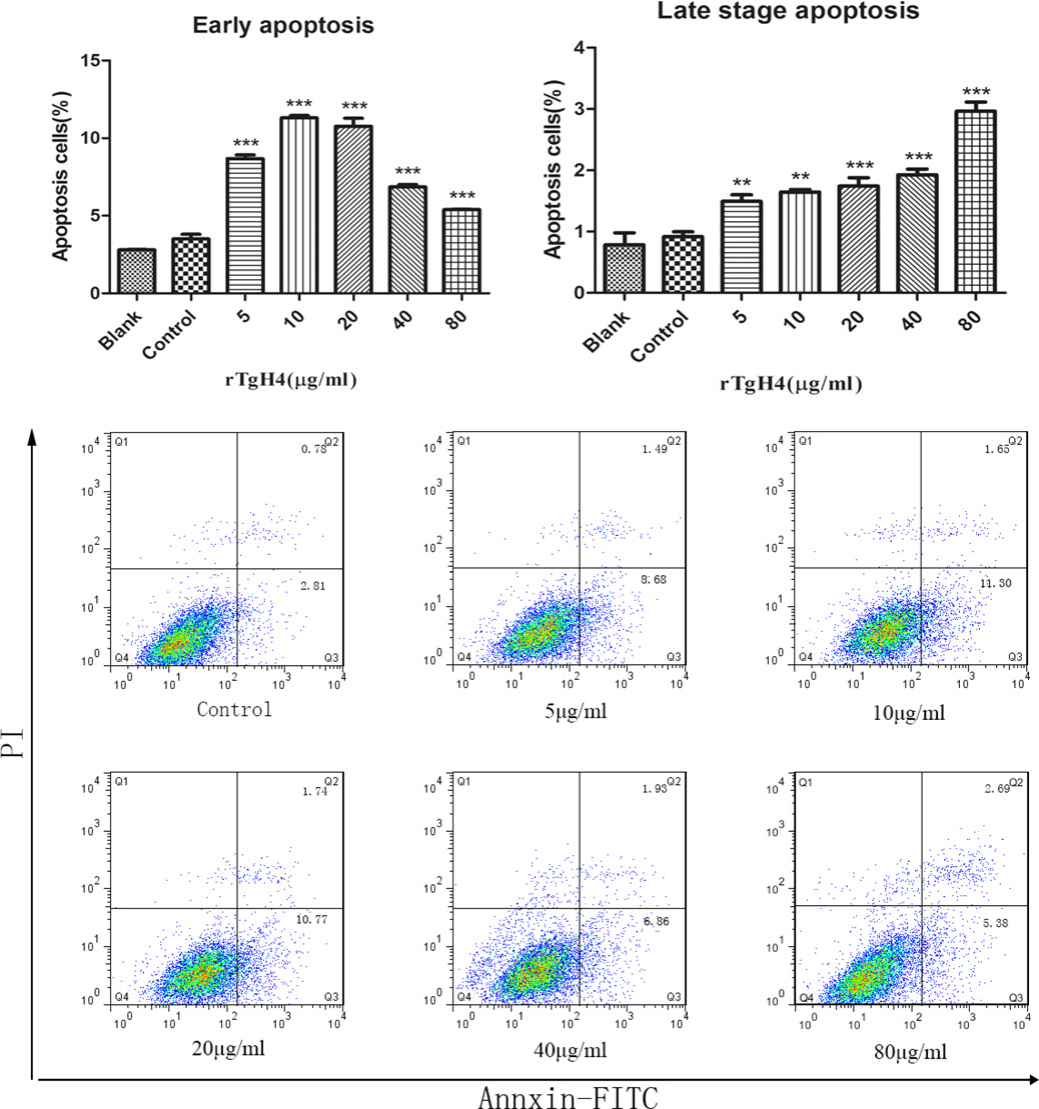
rTgH4 protein induced the apoptosis of the murine macrophages. After incubation with rTgH4, apoptosis was quantified by flow cytometry utilizing an Annexin V‐FITC kit. In the blank group, the cells were treated with PBS, and in the control group, the cells were treated with pET‐32a vector protein. The data were indicative of three individual experiments (**p < 0.01 and ***p < 0.001).

An Annexin V‐FITC kit was utilized to identify the effect of rTgH4 on apoptosis. No effect on macrophages was observed in the blank group treated with PBS and the control group treated with pET‐32a vector protein. The results showed that all of the concentrations of rTgH4 induced both early‐ and late‐stage apoptosis of murine macrophages (Fig. 7).

### rTgH4 inhibited the chemotaxis of murine macrophages

**Figure 8:**
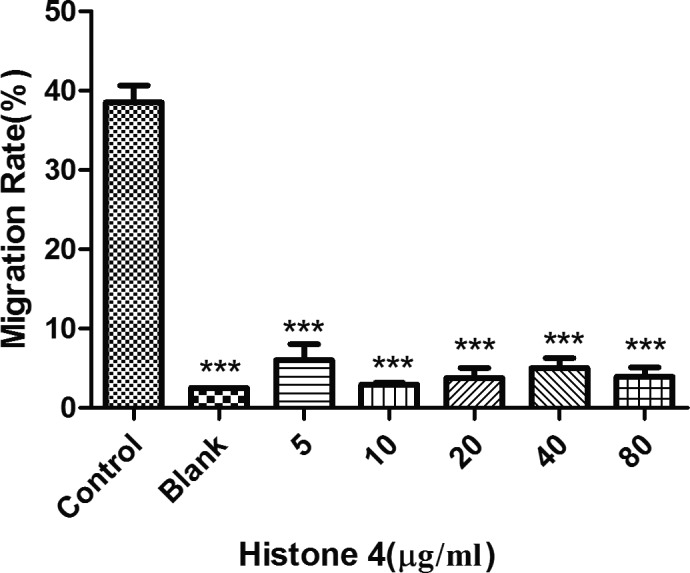
rTgH4 inhibited the chemotaxis of the murine macrophages. Chemoattractants were used to perform the experiments. The untreated cells loaded above the chemoattractants were set as the control groups and the untreated cells without the chemoattractants were set as the blank groups. The data were indicative of three individual experiments (***p < 0.001).

As shown in Fig. 8, in the control groups with chemoattractants, the migration rate of macrophages was about 40%, and in the blank groups without chemoattractants, there was little migration. After treatment with rTgH4, the chemotaxis of the macrophages was inhibited significantly, while the migration rate of the cells was almost the same in the blank groups.

### rTgH4 affected cytokines and NO secretion of macrophages

**Figure 9:**
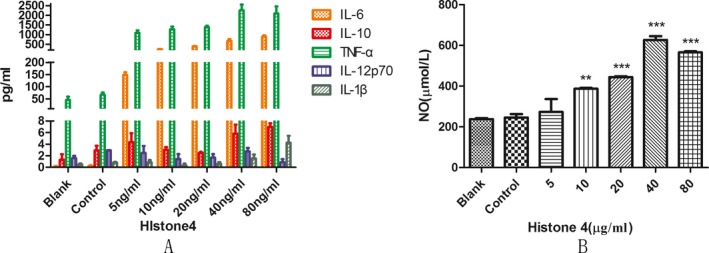
rTgH4 affected the cytokines and NO secretion of macrophages. CBA cell signaling flex sets and a Total Nitric Oxide Kit were utilized to examine the cytokines and NO secretion. In the blank group, the cells were treated with PBS, and in the control group, the cells were treated with pET‐32a vector protein. The data were indicative of three individual experiments (**p < 0.01 and ***p < 0.001).

CBA cell‐signaling flex sets and a Total Nitric Oxide Assay kit were utilized to determine the secretion of cytokines and NO. No effects on secretion by the macrophages were observed in the control groups treated with pET‐32a vector protein. Meanwhile, after treatment with rTgH4, the secretions of NO, TNF‐α, and IL‐6 were promoted in comparison to the control groups. rTgH4 had no significant effect on the secretion of IL‐12 and only 80 μg/ml rTgH4 promoted IL‐10 and IL‐1β secretions of the macrophages (Fig. 9A,B).

## Discussion

Histones are a widely evaluated class of proteins with separate elements that span from being highly conserved in an evolutionary sense to being species specific (Spiker 1976). In this study, the conservation of the *T. gondii* H4 sequence was analyzed and the results showed that in different strains of *T. gondii*, the evolutionarily conserved histone4 sequence were identical. The sequence of histone 4 from *T. gondii* and humans differs in only 5 of 103 amino acids.

Macrophages are a pivotal part of the innate immune system. Macrophages are activated in response to microbial products and host cytokines and release inflammatory/microbicidal factors, which are deterministic factors in protective responses against intracellular pathogens (Wynn et al. 2013). As active scavengers, macrophages affect the clearance of pathogens during the immune response to infection (Lopes et al. 2000). In this study, it was indicated that TgH4 has the capacity of binding macrophages, and after an incubation of macrophages with rTgH4, the proliferation, phagocytosis, apoptosis, chemotaxis, and ability to secrete cytokines and NO were importantly changed. Thus, it can be concluded that rTgH4 has modulating effects on the functions of macrophages.

TLRs are intra/extracellular immune cell receptors that are members of the pathogen recognition receptors family (PRR) and have a vital role in parasite pathogen‐associated molecular pattern (PAMP) recognition and the subsequent immune responses induced against infectious agents, such as *T. gondii* (Zare‐Bidaki et al. 2014). The interaction of TLR with PAMP initiate a variety of immune functions, including migration (Neal et al. 2013), phagocytosis (Anand et al. 2007), and inflammatory cytokine secretion (Schaub et al. 2004). Of all the TLRs involved in innate immune response, TLR4 activates immune responses using either the MYD88 or toll/IL‐1R domain‐containing adaptor inducing IFN‐β (TRIF) pathways, which may induce the expression of pro‐inflammatory cytokines such as IL‐6, TNF‐a (Yamamoto et al. 2003). Furthermore, immune recognition by TLR4 is involved in protective mechanisms against *T. gondii* infection (Furuta et al. 2006). Thus, TLR4 was selected as a representative to assess whether rTgH4 can affect the TLRs level of macrophages. After incubation with rTgH4, the downregulation of the TLR4 level was observed in macrophages in vitro*,* which resulted in the suppression of the immune response. Whether rTgH4 is affecting the other TLRs levels needs to be studied in further research.

Chemokine‐mediated recruitment of macrophages is a necessary function of macrophages to reach the focus of the infection. Directed migration, or chemotaxis, of immune cells is an essential feature of the immune system (Andrew 2001). Without this stage, macrophages’ function as immune regulatory cells or effector cells with phagocytic and cytolytic ability cannot begin (Doherty et al. 1987). In this study, the migration rate of macrophages after incubation with rTgH4 was significantly induced, which means that rTgH4 inhibits the chemotaxis of macrophages, thereby leading to the suppression of the immune response.

*Toxoplasma gondii* is a protozoan that can infect virtually every one of the nucleated cells. Macrophages are vital effector cells of the immune system and host cells for *T. gondii*. Cell proliferation depends on signals to stimulate cell growth and cell division (Joseph et al. 2010). It has been reported that *T. gondii* can suppress host cell proliferation and prompt host cell cycle arrest at the G2/M phase (Brunet et al. 2008). In this study, after treatment with rTgH4, the proliferation of macrophages was suppressed, which resulted in the decrease in immune function.

To exist inside hosts, parasitic protozoans show the capability to regulate host apoptosis pathways to their advantage‐averting apoptosis in host cells that are populated by parasites and inducing apoptosis in host immune cells programmed to assault them (James and Green 2004). In this research, rTgH4 was able to induce both early‐ and late‐stage apoptosis of macrophages, which might inhibit the phagocytosis of macrophages, and provide growth advantage to parasites.

Phagocytosis is a principal component of the body's innate immunity in which macrophages internalize pathogens. After the interaction of TLR with PAMP, phagocytosis and the secretion of molecules were the two generic types of behavior that macrophages exhibited when encountering pathogens (Franken et al. 2016). In this study, the TLR4 level of macrophages was downregulated, while the phagocytosis of the macrophages improved after incubation with rTgH4, so the other mechanisms for regulating phagocytosis played more important roles, thereby promoting the removal of *T. gondii*.

The secretion of cytokines and NO was another important behavior that macrophages exhibited toward pathogens. In this study, five cytokines and NO were selected to study the function of rTgH4 on macrophages. NO has been shown to inhibit the growth and function of a diverse array of infectious disease agents (Wandurska‐Nowak 2004). The type of immune response was regulated by IL‐10 and IL‐12. IL‐10 prevents cytokine synthesis and prompts the advantage of the Th2 involvement reaction by halting the generation of TNF‐α and IL‐6. Nevertheless, IL‐12 assists with the development of a Th1 type response (Matowicka‐Karna et al. 2009; Romagnani 1996; Sher et al. 2003). IL‐1β is an inflammatory cytokine that has been described as a “master regulator” of inflammation since it can activate downstream inflammatory genes (Dinarello 2011). In this study, after incubation with rTgH4, the secretion of NO by macrophages was induced, which leads to the enhancement of the lethal effect of macrophages against *T. gondii*. rTgH4 promoted the secretion of TNF‐α and IL‐6 in murine macrophages and stimulated macrophages to produce IL‐10 and IL‐1β at high concentration (80 μg/ml). The two upregulated cytokines prompt the expression of E‐selectins and integrin ligands on endothelial cells, which in turn permits additional immune cells to infiltrate the site of inflammation (Arango Duque and Descoteaux 2014). In summary, the macrophages’ secretion of cytokines and NO was regulated by rTgH4.

## Conclusion

The *T. gondii* histone 4 was functionally characterized. In addition to the prior understanding of histones as supporters of the chromatin structure, it was indicated that TgH4 can impact the function of macrophages by attaching to the cells. It showed that the TgH4 protein could help hosts to eradicate *T. gondii* through promoting phagocytosis of macrophages and regulating the secretion of NO, IL‐6, and TNF‐α. Meanwhile, the regulation of TLR4 level, chemotaxis, and apoptosis and the proliferation of macrophages were inhibited after incubation with rTgH4, thereby leading to the suppression of the immune response that can be beneficial in the survival and reinfection of *T. gondii*. All these results indicated that TgH4 was involved in the modulation of murine Ana‐1 macrophage functions. Given that the macrophages used in this study were passaged cells, the detailed functions of TgH4 on primary cells, along with the potential roles of TgH4 in the parasite–host interactions in vivo, need to be further investigated.
